# Whole-genome sequencing reveals nosocomial *Clostridioides difficile* transmission and a previously unsuspected epidemic scenario

**DOI:** 10.1038/s41598-019-43464-4

**Published:** 2019-05-06

**Authors:** Sergio García-Fernández, Martinique Frentrup, Matthias Steglich, Aitor Gonzaga, Marta Cobo, Nieves López-Fresneña, Javier Cobo, María-Isabel Morosini, Rafael Cantón, Rosa del Campo, Ulrich Nübel

**Affiliations:** 1 Servicio de Microbiología, Hospital Universitario Ramón y Cajal, and Instituto Ramón y Cajal de Investigación Sanitaria (IRYCIS), Madrid, Spain; 2 Red Española de Investigación en Patología Infecciosa (REIPI), Madrid, Spain; 3 Leibniz Institute DSMZ, Braunschweig, Germany; 4 German Center of Infection Research (DZIF), Braunschweig, Germany; 5 Servicio de Medicina Preventiva, Hospital Universitario Ramón y Cajal, Madrid, Spain; 6 Servicio de Enfermedades Infecciosas, and Instituto Ramón y Cajal de Investigación Sanitaria (IRYCIS), Madrid, Spain; 7 Braunschweig Integrated Center of Systems Biology (BRICS), Technical University, Braunschweig, Germany

**Keywords:** Clinical microbiology, Disease prevention

## Abstract

To trace the routes and frequencies of transmission of *Clostridioides difficile* in a tertiary-care hospital in Madrid (Spain), we sequenced the genomes from all *C*. *difficile* isolates collected over 36 months (2014–2016) that were indistinguishable from any other isolate by PCR ribotyping. From a total of 589 *C*. *difficile* infection cases, we cultivated and PCR-ribotyped 367 *C*. *difficile* isolates (62%), of which 265 were genome-sequenced. Based on close relatedness of successively collected isolates (≤2 SNPs difference in their genomes), whole-genome sequencing revealed a total of 17 independent, putative transmission clusters, caused by various *C*. *difficile* strains and each containing 2 to 18 cases, none of which had been detected previously by standard epidemiological surveillance. Proportions of linked isolates varied widely among PCR ribotypes, from 3% (1/36) for ribotype 014/020 to 60% (12/20) for ribotype 027, suggesting differential aptitudes for nosocomial spread. Remarkably, only a minority (17%) of transmission recipients had direct ward contact to their presumed donors and specific *C*. *difficile* genome types frequently went undetectable for several months before re-emerging later, suggesting reservoirs for the pathogen outside of symptomatic patients. Taken together, our analysis based on genome sequencing suggested considerable within-hospital epidemic spread of *C*. *difficile*, even though epidemiological data initially had been inconspicuous.

## Introduction

The anaerobic bacterium *Clostridioides difficile* [formerly called *Clostridium difficile*]^1^ is the leading cause of antibiotic-associated diarrhoea^2^. Incidence rates of *C*. *difficile* infection (CDI) among patients in healthcare institutions in Europe range between 0.7 and 28.7/10,000 patient-bed days^3^, and the incidence is similar in the USA^2^. Genotyping of *C*. *difficile* isolates facilitates the understanding of CDI epidemiology by tracking the emergence and spread of diverse strains. Presently, the most commonly used technique for genotyping *C*. *difficile* is PCR ribotyping, which charts length variation of spacer sequences in ribosomal RNA operons^4,5^. For example, ribotype 027 represents a *C*. *difficile* lineage that has caused several large-scale outbreaks in hospitals in North America and Europe since the early 2000s, triggering an increased awareness about CDI incidence and severity^6^. Sporadic cases of ribotype 027 have been recorded in Spain since 2007^7^, but it has only been recently that an outbreak caused by this ribotype was reported^8^.

Compared to PCR ribotyping and other genotyping methods, whole-genome sequencing has provided increased discriminatory power and more detailed insights into patterns of *C*. *difficile* spread at both, local^9–11^ and international scales^12–14^. In hospitals in the UK, only a minority of *C*. *difficile* isolates from CDI patients were sufficiently closely related to previously collected isolates to make acquisition through transmission from other CDI cases plausible^10^. This result was unexpected, because the majority of CDI cases commonly were considered healthcare-associated based on surveillance definitions^15^. Apart from CDI patients, however, other potential sources for *C*. *difficile* spread were not investigated in that paper^10^, including asymptomatic carriers^16–18^, colonized staff, or environmental contamination^19–21^. Furthermore, the epidemiology of CDI may be strain-dependent, as genome sequences from *C*. *difficile* isolates collected across Europe suggested distinct spreading patterns for different clonal lineages^12^. While some healthcare-associated ribotypes (027, 001) showed region-specific phylogenetic clustering, others (e.g., 078, 014, 020) appeared to spread effectively over longer distances, possibly associated with the food chain and community acquisition^12^.

In this study, we investigated the molecular, genomic epidemiology of *C*. *difficile* in a tertiary-care hospital in Madrid, Spain. We used PCR ribotyping and whole-genome sequencing of *C*. *difficile* isolates collected from CDI patients over three years (2014 and 2016), to trace routes and frequencies of transmission and to monitor therapeutic success, including therapy by faecal microbiota transplantation. Based on whole-genome sequencing data, we discovered an epidemic situation, which had not been suspected on the basis of epidemiological data alone.

## Methods

### Study design

The study was conducted from January 2014 to December 2016 in a 1,100-bed tertiary-care university hospital in Madrid (Spain) providing medical care for approximately 550,000 inhabitants. Stool samples from all patients with diagnosis of diarrhoea caused by *C*. *difficile* (as described below) during this period were recruited. Information on dates and length of admission, hospitalization wards, and previous admissions was recovered from the Microbiology Department database and from clinical charts. The ethical committee of Ramón y Cajal University Hospital approved the study (no. 266-17) and decided that informed consent from patients was not required, since bacterial isolates were analysed exclusively, in order to investigate and prevent pathogen spread within the hospital. No tissue samples were collected from patients and data were anonymized. All methods were performed in accordance with relevant guidelines and regulations.

A CDI case was considered when a patient (two years or older) presented compatible clinical symptoms (i.e., two or more unformed stools in <24 h) with a positive laboratory assay for *C*. *difficile* toxins in stool (see below). A new case in each patient was defined if the CDI episode was the first one for this patient or if it occurred more than eight weeks after a previous episode^22^. The three-step diagnostic algorithm was applied for the detection of toxigenic *C*. *difficile* in faecal samples based, first, on enzyme immunoassays (EIAs) detecting glutamate dehydrogenase (GDH) (C Diff Quik Chek, Techlab, Blacksburg VA, USA), second, on toxins A/B detection (TOX A/B Quik Chek, Techlab, Blacksburg VA, USA) and, third, on PCR amplification for the *tcd*B gene (BD MAX Cdiff assay, BD Diagnostic, Franklin Lakes, NJ, USA). In addition, untreated stool samples were cultured on *C*. *difficile* ChromID agar (bioMérieux, Marcy l’Etoile, France), and after 48 h of incubation at 37 °C in anaerobic conditions, suspicious colonies were identified by MALDI-TOF MS (Bruker-Daltonics, Bremen, Germany). Antimicrobial susceptibilities to metronidazole, moxifloxacin, tigecycline and vancomycin of randomly selected *C*. *difficile* isolates were tested by using gradient strips (Etest, bioMérieux, Marcy L’Etoile, France) according to the manufacturer’s recommendations and applying MIC breakpoints recommended by EUCAST (http://www.eucast.org/clinical_breakpoints/).

For patients diagnosed with CDI, contact precautions were implemented in our hospital since 2013, including the isolation of CDI patients in single rooms with a private bathroom and the use of barrier precautions (i.e., disposable gown and gloves at the entrance). Cleaning procedures included disinfection with hypochlorite (2,500 ppm available chlorine).

### Epidemiological analyses and surveillance definitions

CDI were classified as: (i) healthcare facility-onset (HO); (ii) community-onset, healthcare facility-associated (CO-HCFA); (iii) community-associated (CA); and (iv) indeterminate, according to published guidelines^22,23^. HO and CO-HCFA cases together were considered healthcare-associated (HA) CDI.

According to our internal protocols, a CDI outbreak is suspected when three or more new cases of CDI occur on the same medical ward within seven days, or two or more cases within seven days on intensive care units. In an outbreak setting, we immediately alert the healthcare personnel working in the affected area and reinforce the infection control measures as described above.

### Toxin gene analysis and PCR ribotyping

Presence of *tcd*A, *tcd*B, *tcd*C, *cdt*A and *cdt*B genes were tested by PCR in all cultured isolates according to previously described protocols^24^. *tcdC* deletions were characterized by Sanger sequencing using an ABI PRISM 3100 Genetic Analyzer (Applied Biosystems, Waltham, MA, USA) and BLASTn comparisons to the Nucleotide Collection database at https://blast.ncbi.nlm.nih.gov. PCR ribotypes of all isolates were determined by using a PCR protocol designed by Bidet *et al*.^25^. The size of DNA fragments was determined using an ABI PRISM 3100 Genetic Analyser apparatus and ribotypes were assigned using the Webribo database (https:/webribo.ages.at/).

### Faecal microbiota transplantation (FMT)

Patients from our hospital with at least three episodes of CDI were selected for FMT, according to internal protocols and following published guidelines^26^. In addition, patients from other institutions were ambulatory admitted to our hospital specifically for FMT. Faecal donors were usually selected among the patient’s relatives, and if no donor could be designated, we used anonymous faecal samples from other donors. Donors had to pass a complete analytic and clinical evaluation to be authorized. FMT was performed by colonoscopy, instilling 50 to 100 grams of faeces dissolved in 500 ml H_2_O into the cecum. Carriage of *C*. *difficile* was checked by microbiological culture from faecal samples collected about one month after FMT.

### Whole genome sequencing

From each bacterial isolate, genomic DNA was extracted by using the DNeasy Blood & Tissue kit according to the manufacturer’s protocol (Qiagen). Illumina sequencing libraries were prepared as described previously^27^ and sequenced on an Illumina NextSeq 500 machine using a Mid-Output kit (Illumina) with 300 cycles. Sequencing reads were mapped to the reference genome sequence from *C*. *difficile* strain R20291 (ribotype 027; sequence accession number FN545816, European Nucleotide Archive) by using BWA-MEM^28^ (v0.7.12) and sequence variation was detected by applying VarScan2^29^ (v2.3) as reported previously^27^. Sequence variation likely generated by recombination was detected through analysis with ClonalFrameML^30^ (v1.11) and removed prior to determination of pairwise sequence distances^11^ and to construction of maximum-likelihood phylogenetic trees with PhyML, implemented in Seaview 4 (http://doua.prabi.fr/software/seaview). For calculating proportions of putative transmissions among CDI cases, we considered transmission recipients from April 2014 to December 2016 only, excluding a run-in period of three months similar to a recently published protocol^11^, because transmission sources for CDI during the first three months of the study period might have been from 2013 and hence not included in the dataset. All genome sequencing data was submitted to the European Nucleotide Archive (www.ebi.ac.uk/ena) under study number PRJEB28391.

## Results

### Epidemiology

During the 36 months of the study, 9,335 faecal samples from diarrhoeic patients were tested for *C*. *difficile* in our institution (Table 1). A total of 735 of these samples tested *C*. *difficile* toxin-positive, leading to the identification of 589 new CDI cases, corresponding to 4.3 HO cases per 10,000 patient days (Table 1). Of note, samples from 226 of these CDI cases (45%) had tested positive in GDH-EIA and toxin-PCR assays only, yet were toxin-EIA negative. The majority (72%) of CDI cases were classified as healthcare-associated according to surveillance definitions (53% HO, 19% CO-HCFA), whereas 25% of cases were community-associated and 4% were indeterminate (Table 1)^23^. For HO, a mean period of 12.9 days (range, 2 to 116) passed from admission to the development of CDI, and for CO-HCFA, an average of 13.9 days (range, 2 to 99 days) passed from discharge to CDI (Table 1). Inpatients with HO were admitted in 33 different medical departments in the hospital, and the majority of cases occurred in the departments for Internal Medicine (25%), Gastroenterology (12%), General Surgery (12%), Oncology (7%), and, less frequently, in Traumatology, Nephrology, Haematology, and Infectious Diseases with around 5% of HO cases each (data not shown).

**Table 1:** Diagnostic and epidemiological data.

	2014	2015	2016	Total
^a^CDI analyses	2,613	3,196	3,526	9,335
Toxin test positive (%)	203 (7.8%)	235 (7.3%)	297 (8.4%)	735 (7.8%)
New Cases	168	193	228	589
Recurrences (%)	22.0%	23.3%	24.6%	23.3%
Age	67.6 (2–95)	68.2 (2–98)	65.7 (2–92)	67.2 (2–98)
Females (%)	64.3%	58.5%	50.4%	57.7%
HO (%)	93 (55.4)	104 (53.9)	110 (48.2)	307 (52.5)
CO-HCFA (%)	35 (20.8)	32 (16.6)	43 (18.9)	110 (18.8)
CA (%)	35 (20.8)	49 (25.4)	63 (27.6)	147 (24.6)
Indeterminate	5 (3.0)	8 (4.1)	12 (5.3)	25 (4.1)
^b^HO rate	3.8	4.3	4.7	4.3
^c^CO-HCFA rate	1.1	1.0	1.3	1.1
HO average length of stay	25.2 (2–120)	21.6 (4–101)	25.1 (3–116)	23.9 (2–116)
HO days from admission to CDI	12.3 (2–68)	10.6 (2–83)	15.8 (2–99)	12.9 (2–99)
CO-HCFA days from discharge to CDI	12.9 (3–30)	15.4 (2–29)	13.5 (2–30)	13.9 (2–30)

^a^Based on a three step algorithm (i.e., glutamate dehydrogenase-EIA plus toxin-EIA, confirmed by toxin-gene PCR).

^b^HO as the number of cases per 10,000 patient-days.

^c^CO-HCFA as the number of cases per 1,000 patient admissions.

### PCR ribotype diversity

**Figure 1:**
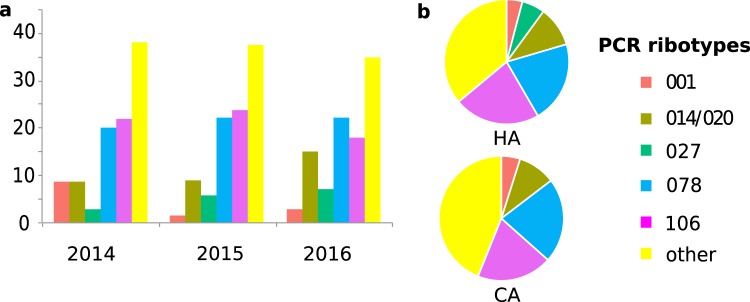
(**a**) Distribution of PCR ribotypes during the three years of the study and **(b)** proportions of PCR ribotypes among HA and CA.

*Clostridioides difficile* isolates from 367 new CDI cases (62% of 589 CDI cases) were successfully cultivated and PCR-ribotyped. All isolates carried the genes encoding enterotoxins A and B, whereas positive PCR amplification of the binary toxin *cdt*A/*cdt*B genes was observed in 106 isolates (29%). The 367 isolates were affiliated to 96 different PCR ribotypes, indicating high genetic diversity among our *C*. *difficile* population. However, the five most prevalent PCR ribotypes accounted for 63% of all isolates, with ribotypes 078 and 106 each causing around 20% of CDI cases throughout the three-year period of the study (Fig. 1). Of note, ribotype 027 had not been detected among 196 *C*. *difficile* isolates collected in our hospital between 2009 and 2013 (data not shown). During the study period (2014–2016), there was a notable increase of PCR ribotype 027 prevalence with concomitant decrease of ribotype 001 (Fig. 1). Proportions of major ribotypes were similar among HA and CA, except that ribotype 027 was not found among CA (Fig. 1).

### Antimicrobial drug susceptibilities

Antibiotic susceptibilities were tested in five isolates from each of the major PCR ribotypes (total number of strain tested *n* = 25). Resistance to metronidazole or vancomycin, routinely used for CDI treatment, was not detected, and tigecycline resistant isolates were not observed either (Table 2). In contrast, the majority of tested isolates were resistant to moxifloxacin, except for those affiliated to PCR ribotype 014/020 (Table 2).

**Table 2:** Antimicrobial susceptibilities of *C*. *difficile* isolates.

PCR ribotype (n)	Vancomycin		Tigecycline		Metronidazole		Moxifloxacin	
	% S^a^	MIC^b^ range	% S	MIC range	% S	MIC range	% S	MIC range
001 (5)	100	0.19–0.5	100	≤0.016–0.023	100	0.064–0.19	20	0.5–>32
014/020 (5)	100	0.25–0.38	100	≤0.016	100	0.032–0.125	100	0.125–0.75
027 (5)	100	0.016–0.5	100	≤0.016–0.023	100	0.125–0.25	0	>32
078 (5)	100	0.25–0.5	100	≤0.016–0.023	100	0.032–0.047	0	>32
106 (5)	100	0.125–0.5	100	≤0.016–0.023	100	0.064–0.125	40	0.5–>32

^a^% S, proportion of susceptible isolates (%); ^b^MIC, minimum inhibitory concentration (mg/L).

### Genomic relatedness

We sequenced the genomes from all 265 *C*. *difficile* isolates affiliated to PCR ribotypes that were represented by at least two isolates. The resulting data provided higher discriminatory power than PCR ribotyping and at the same time enabled analyses of phylogenetic relationships among isolates (Suppl. Figs S1–S6). Recombination-corrected maximum-likelihood phylogenetic trees indicated that subtypes to the canonical PCR ribotypes (provided by the Webribo database on basis of subtle differences in electropherograms) generally did not represent phylogenetically coherent groupings (Suppl. Figs S1–S6). For example, three of four ‘subtypes’ distinguished from ribotype 078 on the basis of single-band differences (078ecdc, 078/1, 078/2, 126) did not cluster phylogenetically but were scattered across the 078 tree (Suppl. Fig. S3). Similarly, isolates with ribotypes 106 and 500, distinguished by a single band in PCR ribotyping, did not cluster in separate phylogenetic clades on the basis of genome sequence variation (Suppl. Fig. S4). For simplicity, in the following we will therefore refer to these two groups of isolates as being affiliated to ribotypes 078 and 106, respectively. Ribotypes 404, 076 and 591, in the genome-based phylogeny all have positions nested within the clade of ribotype 014/020 (Suppl. Fig. S5). Further, genome-based phylogenetic analysis indicated that an isolate displaying a novel PCR ribotype (AI-33) was related to ribotype 027 (Suppl. Fig. S2).

Among 367 *C*. *difficile* isolates collected April 2014 to December 2016, 41 (11%) were closely related (i.e. displaying ≤2 SNPs difference in their genomes) to other isolates that had been collected in our hospital less than 90 days before (Suppl. Fig. S13). Between these linked CDI cases, direct transmission may be considered plausible, using previously proposed thresholds^11^. Only seven CA cases (8% of 83 that had been ribotyped) yielded isolates that were linked genetically and temporally (i.e., ≤2 SNPs, ≤90 days) to previous isolates from the hospital, suggesting these infections had resulted from transmission in the hospital rather than the community. In contrast, 34 (12%) of 284 isolates from HA cases were linked to previous isolates. Strikingly, proportions of linked isolates varied widely among PCR ribotypes, from 3% for ribotype 014/020 to 60% for ribotype 027 (Table 3). After correcting for incomplete sampling (62%)^11^, the proportion of linked cases was estimated as 19% overall.

**Table 3:** Proportion of isolates linked to a previous case (≤2 SNPs, ≤90 days) by PCR ribotype, April 2014 to December 2016.

PCR Ribotype	^a^Isolates	^b^Putative transmissions
001	15	5 (33%)
014/020	36	1 (3%)
027	20	12 (60%)
078/126	59	7 (12%)
106	59	14 (24%)
446	4	2 (50%)
^c^Other	174	0 (0%)
Total	367	41 (11%)

^a^One isolate per CDI case.

^b^Number of genomes linked to a previous case (≤2 SNPs, ≤90 days).

^c^Including 102 singletons and the following ribotypes with multiple isolates (number of isolates): 003 (7), 005 (7), 010 (2), 017 (3), 023 (3), 026 (2), 029 (2), 042 (2), 049 (2), 056 (4), 070 (4), 087 (4), 209 (5), 412 (2), 434 (2), 449 (5), 551 (2), 591 (3), 592 (4), 610 (3), AI-78 (2), AI-83 (2).

**Figure 2:**
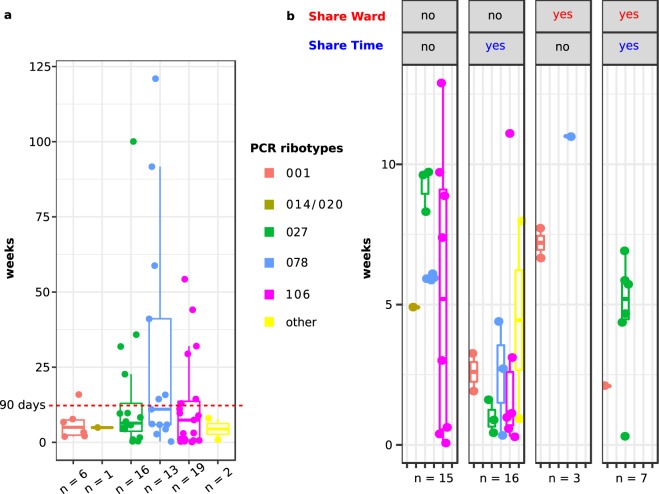
Time intervals between isolates from putative transmission events. Each dot represents one pair of closely related *C*. *difficile* isolates (i.e., ≤2 core-genome SNPs). Horizontal lines indicate the median values, boxes indicate the first and third quartiles, respectively, and vertical lines indicate the maximum and minimum values. Colours indicate the PCR ribotypes. **(a)** 57 putative transmission events. Isolates were linked genetically, but 30% of closely related isolates had been recovered more than 90 days apart. **(b)** Stratification based on information whether CDI patients had stayed at the hospital during the same time or on the same medical ward, respectively. Patients associated with 17% of putative transmission events shared time on the same ward, and patients from 39% of putative transmission events shared time at the hospital, but on separate wards. Only isolates that were linked genetically and temporally (i.e., ≤2 SNPs, ≤90 days) are shown.

Isolates from 21 additional CDI cases were closely related (≤2 SNPs) to previous isolates, yet with time intervals longer than 90 days (Fig. 2A), and in six of these cases, minimum time intervals to close relatives were even longer than one year. Supplementary Figs S7 to S12 illustrate 62 possible transmission events towards CDI patients, considering close genetic relatedness (≤2 SNPs) and the shortest possible time intervals between isolate recovery dates. Altogether 17 putative transmission clusters were detected, each involving two to 18 patients (Suppl. Figs S7 to S12). Remarkably, among the 41 linked cases, only ten pairs of patients (24%) had been admitted on the same ward, and only seven (17%) had been on the same ward during the same time (Fig. 2B). In contrast, 18 (44%) linked cases did not share any time in the hospital with their presumptive sources of transmission, suggesting transmission is common without direct contact between symptomatic patients (Fig. 2B). Specific *C*. *difficile* genotypes were undetectable in the hospital for extended time periods, before closely related isolates (≤2 SNPs) re-emerged again later (Suppl. Figs S7 to S12). These intervals frequently lasted for several months and in some cases even for more than one year (Suppl. Figs S7 to S10). Interestingly, four patients had stayed in the exact same bed as their presumed strain donors, with temporal distances between zero and 100 days, pointing at environmental contamination as a potential reservoir for *C*. *difficile* (Suppl. Figs S1 to S3).

Transmission dynamics were slightly elevated in the Internal Medicine and Gastroenterology wards. While 25% and 12% of HO cases occurred in these two wards, respectively, they accounted for 29% and 17% of the putative transmissions (i.e. 14 and 8 CDI cases on these wards, respectively, were genetically linked to previous cases; Suppl. Figs S7–S13). Further, out of 12 putative transmissions among patients that had been on the same ward, seven occurred on Internal Medicine and three on Gastroenterology wards, respectively. At the same time, Internal Medicine had the highest proportion of CDI cases with PCR ribotype 027 (35%) and the highest proportion of HO (25%).

**Figure 3:**
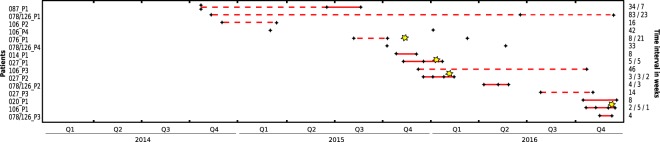
Time lines of patients with two or more *C*. *difficile* isolates. Black diamonds indicate dates of *C*. *difficile* isolation and yellow stars indicate dates of faecal microbiota transplantation. Connecting lines indicate closely related isolates (i.e., ≤2 core-genome SNPs), with solid lines indicating time intervals ≤ eight weeks and dashed lines indicating > eight weeks.

From 15 patients that had two or more episodes of CDI several weeks apart, we included one *C*. *difficile* isolate from each episode for genome sequencing (Fig. 3). In 13 of these cases, all isolates from separate episodes were closely related (≤2 SNPs), demonstrating that those patients had relapses rather than reinfections by another strain. Notably, six (46%) of the presumed relapses had occurred more than eight weeks after the initial episodes (range, 8.1 to 23 weeks), i.e. beyond the currently applied cut-off for surveillance-based detection of CDI relapses^22^.

Three out of four isolates of *C*. *difficile* recovered from faecal samples one week after FMT were genetically closely related (≤2 SNP) to the strains that previously had caused disease (Fig. 3), highlighting the continued colonization of these patients in spite of satisfactory clinical courses.

## Discussion

Among CDI cases that occurred from 2014 to 2016 in a tertiary-care hospital in Madrid, Spain, PCR ribotyping revealed a large diversity of *C*. *difficile* strains commonly found in European settings^31^. Predominant PCR ribotypes included those typically associated with transmission among patients in healthcare facilities (ribotypes 106, 027, 001), and those thought to have different reservoirs, possibly associated with food or environmental contamination (014/020, 078)^12^. Resistance to metronidazole, vancomycin or tigecycline was not found.

Almost 50% of relapses occurred >8 weeks after the initial episodes, and similar intervals had been observed in earlier analyses relying on PCR ribotyping^32,33^ or multilocus sequence typing^34^, and in one previous genome-based examination^9^. While this data lends support to a change of the standard definition of relapse^32^, the number of patients included (15) was limited, and reinfections with identical strains cannot be ruled out entirely in these cases, since the environment around CDI patients may frequently be contaminated with *C*. *difficile* spores^35^.

We observed that a high proportion (3/4) of post-FMT patients were still colonized with the disease-causing strain. Even though this observation was based on a very small number of cases (n = 4), it may warrant follow-up investigations, since it is in stark contrast to a recent publication which reported that asymptomatic carriage after FMT was very rare (2% after one week, 3% after four weeks)^36^.

Lately, the prevalence of ribotype 027 has been declining in some countries, possibly driven more strongly by a reduction of fluoroquinolone antibiotic usage rather than by improved infection control^37^. In Spain, however, ribotype 027 had been uncommon until 2014, when it caused a large outbreak in a hospital in Madrid^8^. In our hospital, ribotype 027 had not been detected prior to September 2014, yet once it had got introduced, its prevalence increased steadily to 7% of all CDI cases in 2016. Genome sequencing indicated that all 027 isolates except one were extremely closely related, consistent with only two imports of 027 into the hospital. At least 60% of 027 infections were caused by transmission between CDI patients, driving continued persistence and spread within the hospital. Further, it is plausible that the emergence of 027 may have caused the concomitant decrease of 001 prevalence, since both strains are resistant to fluoroquinolones and hence may occupy a similar niche, as observed in other locations in the past^38,39^. However, ribotype 106 is another fluoroquinolone-resistant strain that displayed high prevalence (21%) in our hospital and a healthcare-associated transmission pattern. Ribotype 106 has a more restricted international distribution than the former two strains, yet it was reported from hospitals in Spain before^31^. Ribotypes 027 and 106 each caused large and previously unnoticed outbreaks, protracting over the entire study period and involving 17 and 18 patients, respectively (Suppl. Figs S8, S10).

Ribotypes 014/020 and 078 were also highly prevalent in our hospital, even though there was more limited evidence for within-hospital transmission, particularly for ribotype 014/020. Instead, we observed large phylogenetic diversity among these isolates, consistent with numerous independent introductions to the healthcare facility, presumably by colonized patients. Both these strains are globally distributed and reach high prevalence in many regions, including Spain^40^. While their reservoirs and means of spread are not understood, 078 is frequently found in livestock (fattening pigs, in particular), and it has been speculated this strain may spread internationally via the food chain^12^. In contrast to ribotype 014/020, ribotype 078 is frequently fluoroquinolone resistant^41^.

Taken together, our genomic data provides evidence of frequent within-hospital transmission of healthcare-associated ribotypes (ribotypes 027, 001 and 106), in contrast to those strains commonly found in livestock and among CA (ribotypes 078 and 014/20). These results corroborate recent observations of lineage-specific spreading routes across Europe^12^ and further indicate that distinct, associated transmission patterns may also be observed at a local scale. Naturally, the epidemic processes in our hospital were embedded in a larger setting, such that pathogens may have entered and left the institution in association with patients, and therefore we cannot exclude that outbreaks may have extended beyond our institution. While our hospital recruited numerous patients through transfers from other hospitals during the study period, however, we detected only a single patient suffering an infection with *C*. *difficile* ribotype 027 in the second half of 2015 (patient *027_E* in Suppl. Fig. S8), who had previously stayed in another hospital in Madrid where an outbreak with the same PCR ribotype had been ongoing at the time^8^. The ribotype 027 isolate from this patient had a genome that was indistinguishable from those of two other isolates in our hospital, suggesting the two outbreaks may indeed have been interrelated. Elucidating these potential connections at greater detail, however, would require inclusion of genome sequences from *C*. *difficile* isolates from the other hospital.

Strikingly, almost half of the plausible transmission events were predicted between patients that had not shared any time in the hospital. Some of these transmissions might in fact have originated from infected patients that went undetected, either because infections had not been diagnosed due to mild courses of disease or to low sensitivity of the toxin EIA^22^, or because *C*. *difficile* isolates had not been cultured from these CDI cases. Some *C*. *difficile* isolates were lost later when they failed to re-grow from frozen stocks or because cultures got contaminated. However, the sampling strategy and recovery rate were uniform throughout the three years of the study (Suppl. Fig. S13), and CDI cases that were not represented among cultivated *C*. *difficile* isolates were randomly distributed through time. Therefore, the lack of some isolates should not have introduced much bias to our results, and incomplete sampling is unlikely to explain why only 17% of transmission recipients had direct ward contact to their presumed donors. Instead, a significant proportion of transmissions must have occurred either indirectly, e.g. through environmental contamination with *C*. *difficile* spores, or from reservoirs outside of CDI patients, such as asymptomatically colonized patients or staff. Such indirect transmission may also explain the observed long time intervals (i.e. >90 days, with a maximum of 847 days) between isolation dates from a large proportion of presumed sources and recipients. In four extreme cases, CDI patients had occupied the exact same hospital beds as their presumed sources of *C*. *difficile*, albeit two of them had done so 97 and 100 days apart, respectively. This result provides a hint at environmental contamination as a relevant source for *C*. *difficile*. *Clostridioides difficile* spores can stay viable and infective in the inanimate environment for long time periods and their efficient inactivation is challenging. While the quantitative contribution of spore intake to hospital-onset CDI has not been assessed, *C*. *difficile* has frequently been cultured from surfaces in hospital rooms^19^. Moreover, prior room occupancy by a CDI patient was shown to be a risk factor for CDI acquisition by subsequent patients^42^. Due to frequent movements of patients, acquisition of *C*. *difficile* spores may also occur from contaminated surfaces outside of patient rooms. For example, contact of CDI patients with central diagnostic equipment (i.e., a computed tomography scanner) was recently found to increase the odds of subsequent users to also develop CDI^20^. Consequently, the thoroughness of cleaning both rooms and equipment was recommended to be improved, including repetitive training of cleaning personnel and regular quality control^20,21^. In addition, around 8% of patients may be asymptomatically colonized upon hospital admission^43^ and they clearly contribute to transmission^44^. Indeed, previous investigations using highly discriminatory molecular typing showed that CDI cases were equally frequently linked to asymptomatic carriers as to previous CDI patients^17,45^. Furthermore, ward-level exposure to asymptomatic carriers in a hospital increased the risk of developing CDI by almost two-fold^16^, and identification of *C*. *difficile* carriers at hospital admission (by rectal swabbing and toxin-gene PCR) and isolation through contact precautions reduced the overall CDI rate in an acute care facility by 62%^18^. Even though asymptomatic persons are about 15-fold less likely to transmit *C*. *difficile* than CDI patients, they may substantially contribute to CDI prevalence and spread due to the large size of the reservoir^46^. Colonized hospital staff is another conceivable source for *C*. *difficile*, but its role for transmission of gastrointestinal pathogens in general has been investigated comparatively little. During this study, samples from the environment and from asymptomatic patients or staff members were not available unfortunately, but studies to quantify effects of these potential reservoirs are highly warranted.

In our hospital, we have implemented an epidemiological surveillance system, ensuring that the detection of *C*. *difficile* (and of other relevant nosocomial pathogens, e.g. multidrug resistant bacteria) gets communicated to the Preventive Medicine department immediately (i.e., on the same day), to initiate appropriate control measures in the medical wards in case of a suspected outbreak. However, the epidemiological surveillance data alone had not been sufficient to disclose the extent of continued epidemic spread of *C*. *difficile*.

### Concluding remarks

Exhaustive whole-genome sequencing revealed that various *C*. *difficile* strains had caused altogether 17 independent transmission clusters, each containing 2 to 18 cases, and involving a total of 85 patients. Clinicians, hygiene personnel, and healthcare workers had not been aware of this epidemic situation, because the overall frequency of CDI had remained stable at a low level and isolation measures had been considered suitable to prevent transmission of *C*. *difficile* among patients. The most unexpected result of the present work revealed frequent nosocomial transmission between unrelated patients on separate wards and over time-intervals longer than 90 days. We conclude that in spite of inconspicuous epidemiological data, genome sequencing may be extremely useful to understand the local *C*. *difficile* situation. Hence, prospective bacterial genome sequencing should be considered for institutional preventive policies of hospitals^47^.

## Supplementary information

Supplement

## Data Availability

All genome sequencing data was submitted to the European Nucleotide Archive (www.ebi.ac.uk/ena) under study number PRJEB28391.
