# Supplementary material for: Whole-genome sequencing reveals nosocomial Clostridioides difficile transmission and a previously unsuspected epidemic scenario
Source: Sci Rep. 2019 May 6;9:6959. doi: 10.1038/s41598-019-43464-4 (PMC6502822; doi:10.1038/s41598-019-43464-4)
Supplement: Supplementary file 1 — Supplement [file 41598_2019_43464_MOESM1_ESM.pdf]

SUPPLEMENT

Whole-genome sequencing reveals nosocomial *Clostridioides difficile* transmission and a previously unsuspected epidemic scenario

Sergio García-Fernández[1,2], Martinique Frentrup[3], Matthias Steglich[3,4], Aitor Gonzaga[3], Marta Cobo[1], Nieves López-Fresneña[5], Javier Cobo[2,6], María-Isabel Morosini[1,2], Rafael Cantón[1,2], Rosa del Campo[1,2], Ulrich Nübel[3,4,7,*]

[1] Servicio de Microbiología, Hospital Universitario Ramón y Cajal, and Instituto Ramón y Cajal de Investigación Sanitaria (IRYCIS), Madrid, Spain
[2] Red Española de Investigación en Patología Infecciosa (REIPI), Madrid Spain
[3] Leibniz Institute DSMZ, Braunschweig, Germany
[4] German Center of Infection Research (DZIF), Braunschweig, Germany
[5] Servicio de Medicina Preventiva, Hospital Universitario Ramón y Cajal, Madrid, Spain
[6] Servicio de Enfermedades Infecciosas, and Instituto Ramón y Cajal de Investigación Sanitaria (IRYCIS), Madrid, Spain
[7] Braunschweig Integrated Center of Systems Biology (BRICS), Technical University, Braunschweig, Germany

LEGENDS TO SUPPLEMENTARY FIGURES

Supplementary Figure S1. Phylogenetic relationships among isolates from ribotype 001 contructed with PhyML. Symbols indicate the acquisition of *C. difficile* based on surveillance definitions. Bootstrap values >90% are indicated in the tree.

Supplementary Figure S2. Phylogenetic relationships among isolates from ribotype 027 and AI-33 (isolate CD-17-00124). Symbols and bootstrap values as in Suppl. Figure S1.

Supplementary Figure S3. Phylogenetic relationships among isolates from ribotype 078. Symbols and bootstrap values as in Suppl. Figure S1.

Supplementary Figure S4. Phylogenetic relationships among isolates from ribotype 106. Symbols and bootstrap values as in Suppl. Figure S1.

Supplementary Figure S5. Phylogenetic relationships among isolates from ribotype 014/020. Symbols and bootstrap values as in Suppl. Figure S1.

Supplementary Figure S6. Phylogenetic relationships among isolates from ribotype 446. Symbols and bootstrap values as in Suppl. Figure S1.

Supplementary Figure S7. Putative transmission events connecting ribotype 001 isolates. The figure shows three clusters involving four (red), three (yellow) and two (blue) patients, respectively. Data from all patients that shared closely related *C. difficile* isolates (i. e., ≤2 core-genome SNPs) with other patients are illustrated. Green bars indicate the patients' times of hospital stay and black diamonds indicate dates of *C. difficile* isolation. Connecting lines indicate closely related isolates (i. e., ≤2 core-genome SNPs), with solid lines indicating time intervals ≤90 days and dashed lines indicating >90 days. Asterisks indicate patients with closely related isolates (i. e., ≤2 core-genome SNPs) but not linked to a previous case. Patients *001_B* and *001_F* had occupied the same bed (97 days apart). Acronyms for ward names are as follows: Anaesthesia and Resuscitation Department (ANRH); Angiology and Vascular Surgery (VASH); Cardiology (CAAH); Gastroenterology (GASH); General and digestive surgery (CGDH); Gynaecology (GINH); Haematology (HEMH); Infectious diseases (EINH); Intensive Care Department (MIVH); Internal medicine (MINH); Nephrology (NFRH); Neurology (NRLH); Oncology (ONCH); Otolaryngology (ORAH); Pneumology (NMLH); Plastic Surgery (CPLH); Psychiatry (PQAH); Traumatology (TRAH/TOGH); Urology (UROH). In case the patient stayed in different wards, the underlined ward indicates where the *C. difficile* was isolated.

Supplementary Figure S8. Putative transmission events connecting ribotype 027 isolates. Symbols and lines as in Suppl. Figure S7. The figure shows one cluster involving 17 patients. Patients *027_G* and *027_H* had occupied the same bed (0 days apart) and patients *027_G* and *027_J* had occupied the same bed (100 days apart).

Supplementary Figure S9. Putative-transmission events connecting ribotype 078 isolates. Symbols and lines as in Suppl. Figure S7. The figure represent four clusters, involving 13 (blue), three (red), three (purple) and two (yellow) patients, respectively. Patients *078/126_I* and *078/126_K* had occupied the same bed (50 days apart).

Supplementary Figure S10. Putative-transmission events connecting ribotype 106 isolates. Symbols and lines as in Suppl. Figure S7. The figure shows seven clusters, involving 18 (red), three (olive), two (blue), two (sky blue), two (brown), two (pink) and two (yellow) patients, respectively.

Supplementary Figure S11. Putative-transmission events connecting ribotype 014/020 isolates. Symbols and lines as in Suppl. Figure S7. The figure shows one cluster involving two patients.

Supplementary Figure S12. Putative-transmission events connecting ribotype 446 isolates. Symbols and lines as in Suppl. Figure S7. The figure shows one cluster involving three patients.

Supplementary Figure S13. Flow diagram indicating numbers of analyses.

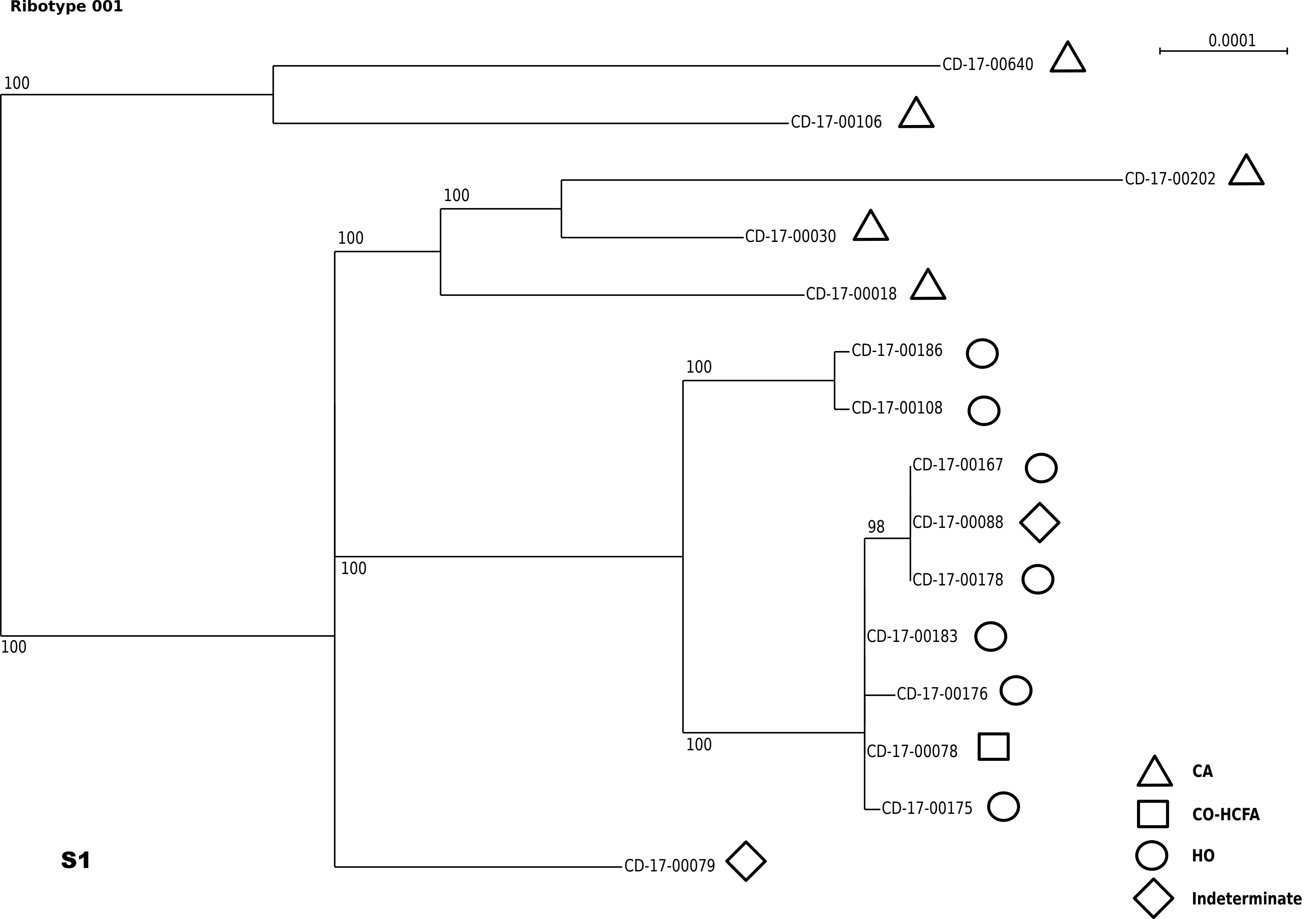
Ribotype 001
0.0001
100
CD-17-00640
CD-17-00106
100
CD-17-00202
100
CD-17-00030
CD-17-00018
100
CD-17-00186
CD-17-00108
CD-17-00167
98
CD-17-00088
CD-17-00178
100
100
CD-17-00183
CD-17-00176
100
CD-17-00078
CD-17-00175
CD-17-00079
CA
CO-HCFA
HO
Indeterminate
S1

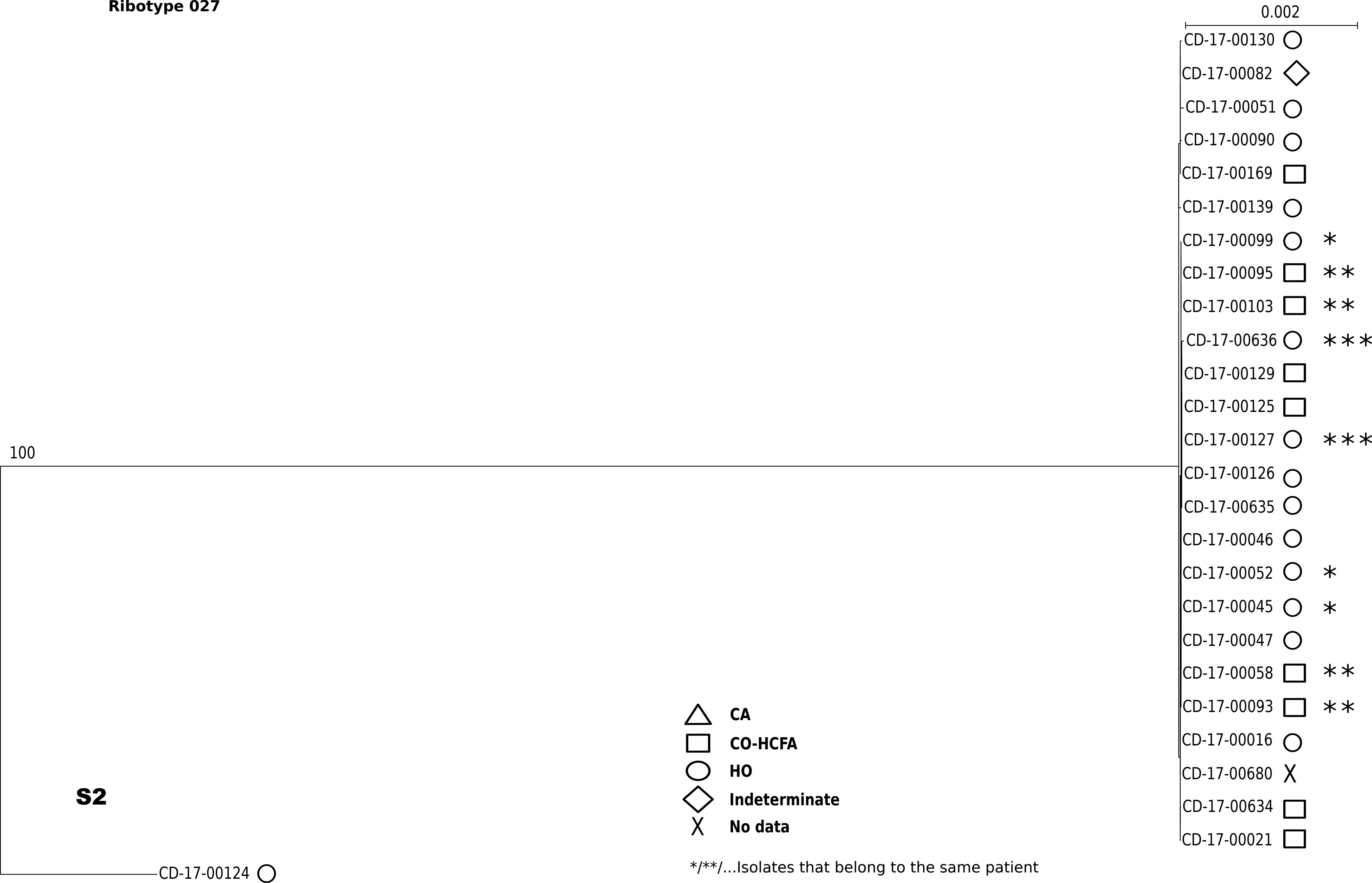

Ribotype 027
0.002
100
CD-17-00130
CD-17-00082
CD-17-00051
CD-17-00090
CD-17-00169
CD-17-00139
CD-17-00099 *
CD-17-00095 **
CD-17-00103 **
CD-17-00636 ***
CD-17-00129
CD-17-00125
CD-17-00127 ***
CD-17-00126
CD-17-00635
CD-17-00046
CD-17-00052 *
CD-17-00045 *
CD-17-00047
CD-17-00058 **
CD-17-00093 **
CD-17-00016
CD-17-00680
CD-17-00634
CD-17-00021
CD-17-00124
S2
CA
CO-HCFA
HO
Indeterminate
No data
*/**/...Isolates that belong to the same patient

Ribotype 078

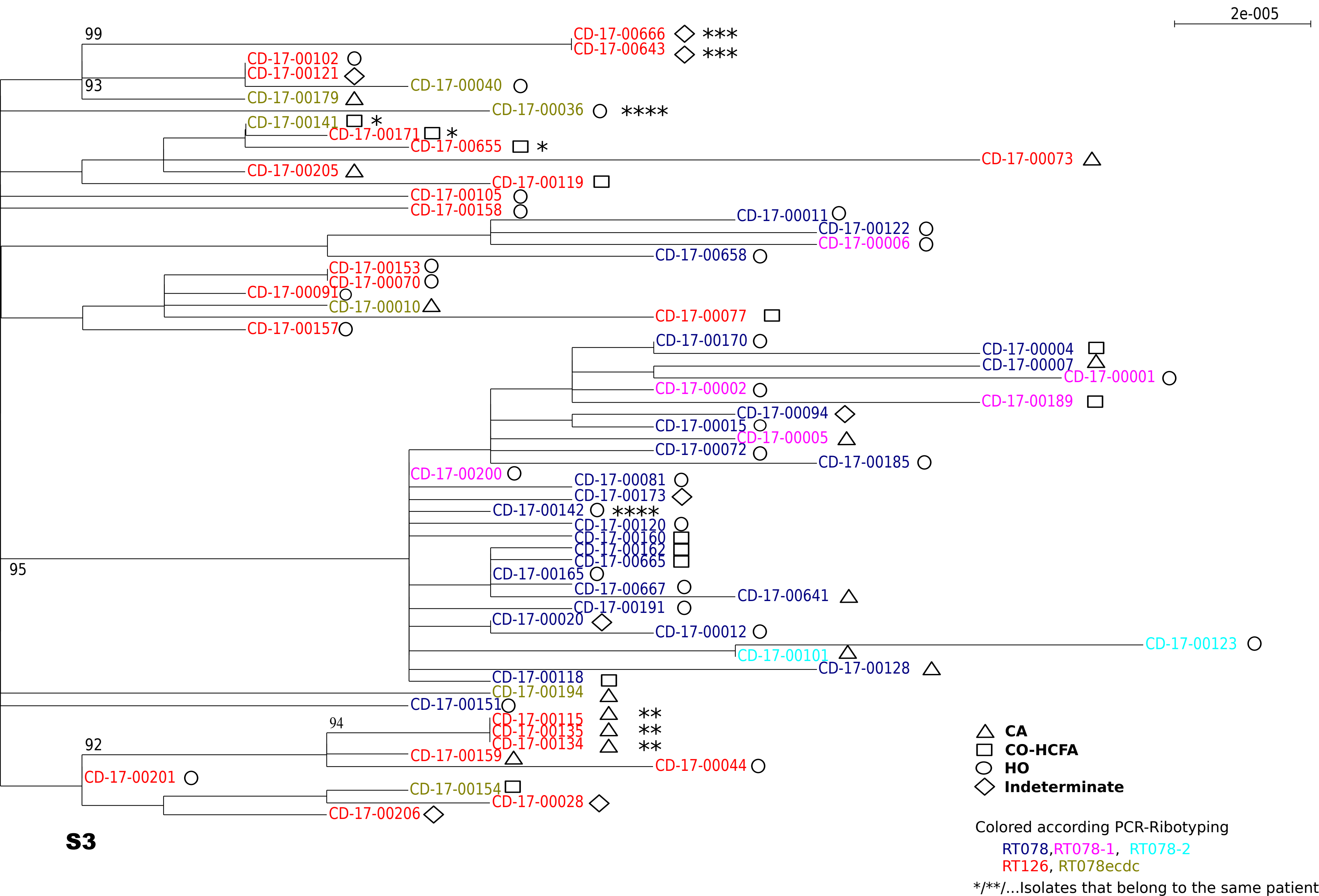

S3

Ribotype 106

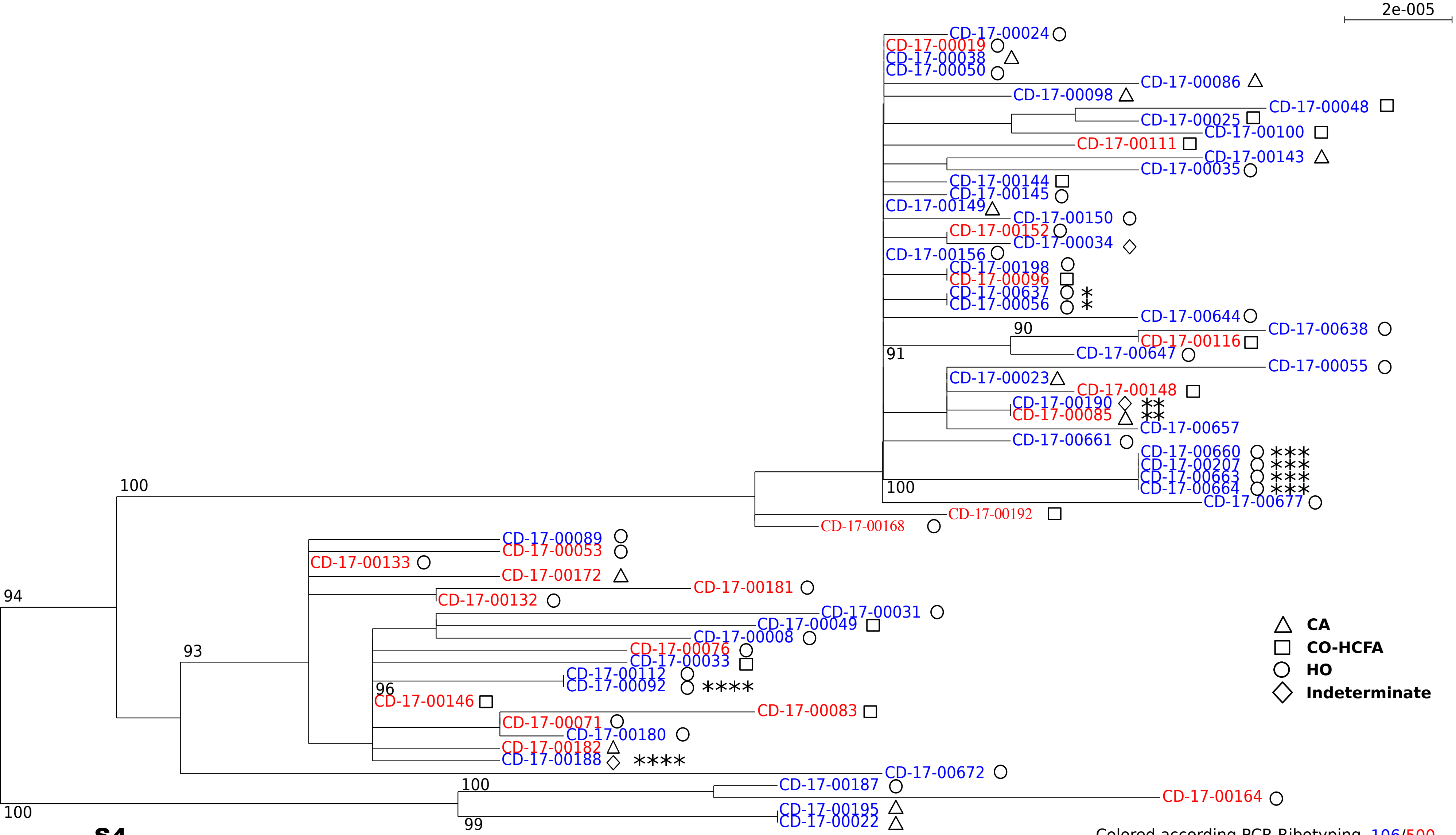

S4

Colored according PCR-Ribotyping 106/500

*/**/...Isolates that belong to the same patient

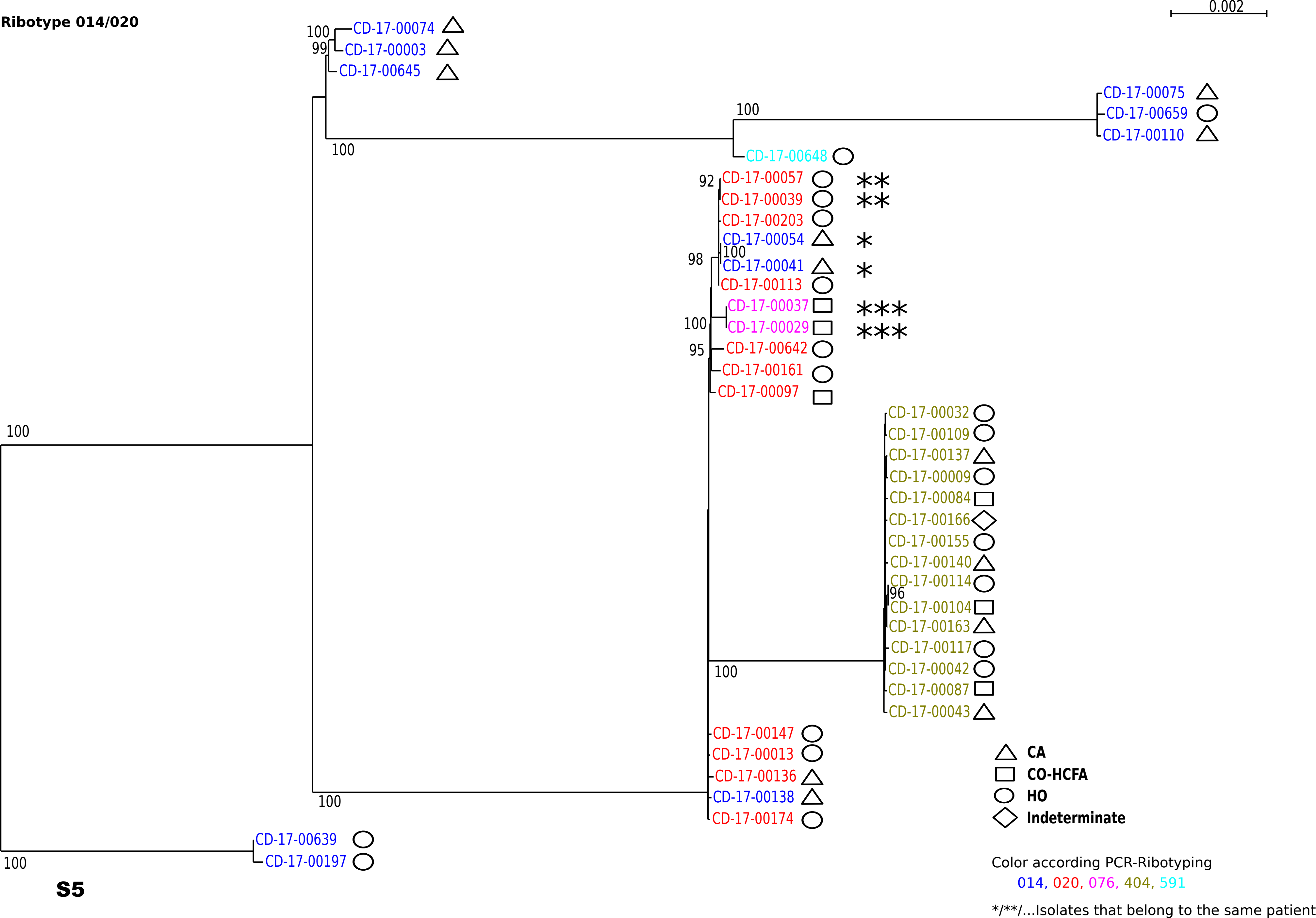
Ribotype 014/020
0.002
CD-17-00074
CD-17-00003
CD-17-00645
CD-17-00075
CD-17-00659
CD-17-00110
CD-17-00648
CD-17-00057 **
CD-17-00039 **
CD-17-00203
CD-17-00054 *
CD-17-00041 *
CD-17-00113
CD-17-00037 ***
CD-17-00029 ***
CD-17-00642
CD-17-00161
CD-17-00097
CD-17-00032
CD-17-00109
CD-17-00137
CD-17-00009
CD-17-00084
CD-17-00166
CD-17-00155
CD-17-00140
CD-17-00114
CD-17-00104
CD-17-00163
CD-17-00117
CD-17-00042
CD-17-00087
CD-17-00043
CD-17-00147
CD-17-00013
CD-17-00136
CD-17-00138
CD-17-00174
CD-17-00639
CD-17-00197
CA
CO-HCFA
HO
Indeterminate
Color according PCR-Ribotyping
014, 020, 076, 404, 591
*/**/...Isolates that belong to the same patient
S5

Ribotype 446

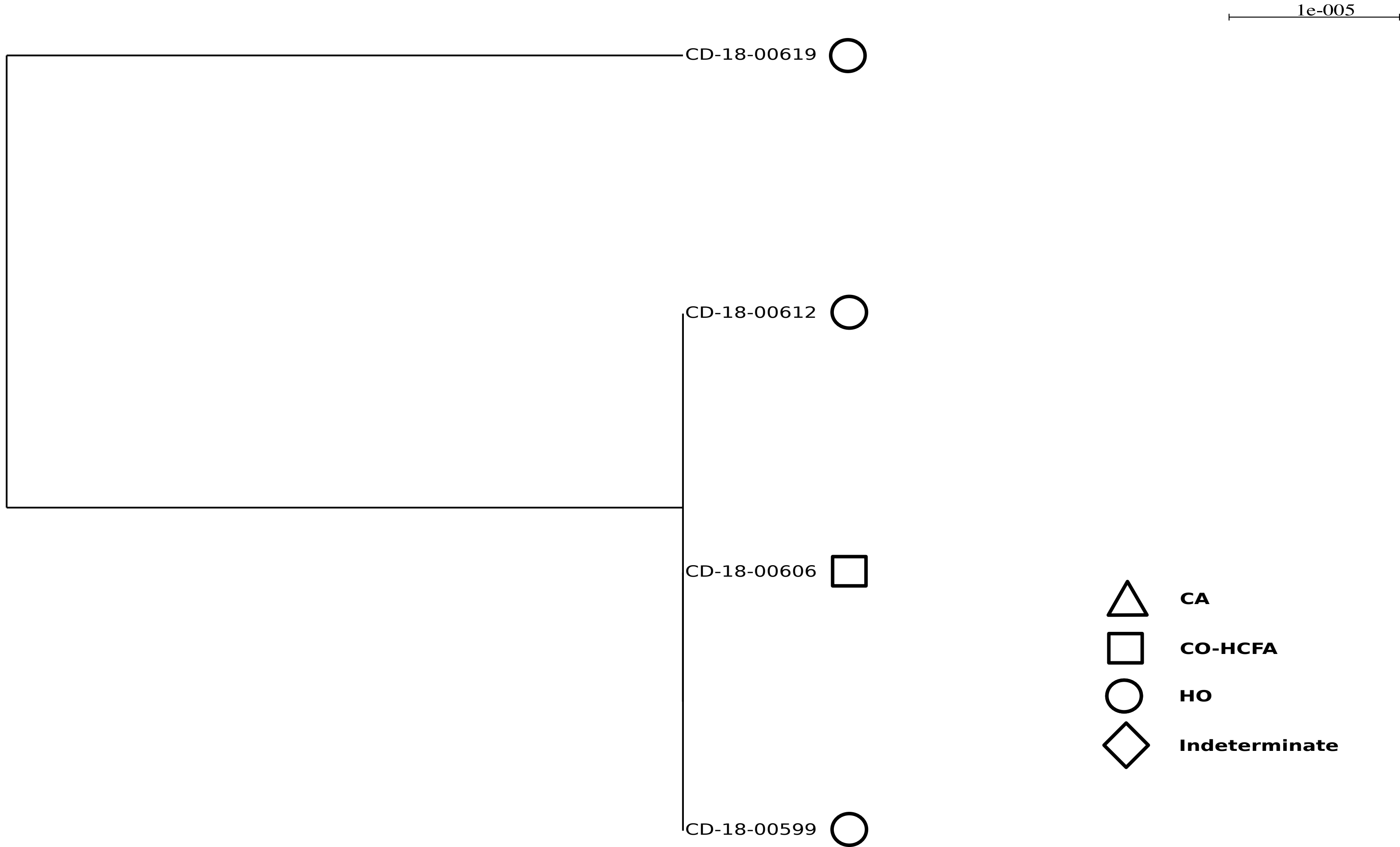

S6

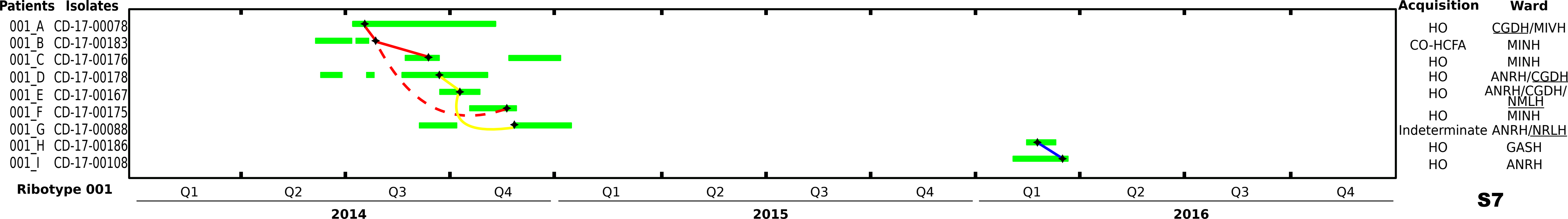
Patients Isolates
001_A CD-17-00078
001_B CD-17-00183
001_C CD-17-00176
001_D CD-17-00178
001_E CD-17-00167
001_F CD-17-00175
001_G CD-17-00088
001_H CD-17-00186
001_I CD-17-00108
Ribotype 001
Q1 Q2 Q3 Q4
2014
Q1 Q2 Q3 Q4
2015
Q1 Q2 Q3 Q4
2016
Acquisition Ward
HO CGDH/MIVH
CO-HCFA MINH
HO MINH
HO ANRH/CGDH
HO ANRH/CGDH/NMLH
HO MINH
Indeterminate ANRH/NRLH
HO GASH
HO ANRH
S7

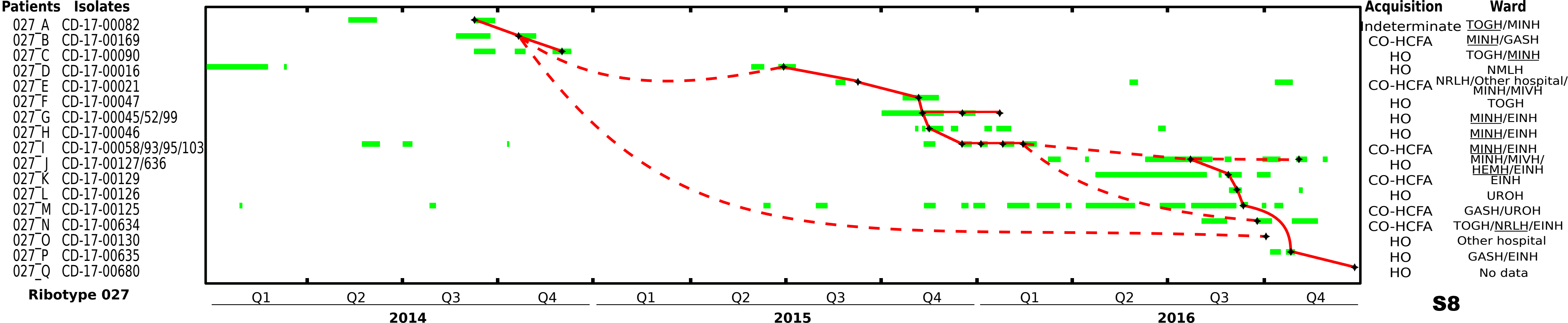
Patients
Isolates
027_A CD-17-00082
027_B CD-17-00169
027_C CD-17-00090
027_D CD-17-00016
027_E CD-17-00021
027_F CD-17-00047
027_G CD-17-00045/52/99
027_H CD-17-00046
027_I CD-17-00058/93/95/103
027_J CD-17-00127/636
027_K CD-17-00129
027_L CD-17-00126
027_M CD-17-00125
027_N CD-17-00634
027_O CD-17-00130
027_P CD-17-00635
027_Q CD-17-00680
Ribotype 027
Q1 Q2 Q3 Q4 Q1 Q2 Q3 Q4 Q1 Q2 Q3 Q4
2014
2015
2016
Acquisition
Ward
Indeterminate TOGH/MINH
CO-HCFA MINH/GASH
HO TOGH/MINH
HO NMLH
CO-HCFA NRLH/Other hospital/MINH/MIVH
HO TOGH
HO MINH/EINH
HO MINH/EINH
CO-HCFA MINH/EINH
HO MINH/MIVH/HEMH/EINH
CO-HCFA EINH
HO UROH
CO-HCFA GASH/UROH
CO-HCFA TOGH/NRLH/EINH
HO Other hospital
HO GASH/EINH
HO No data

S8

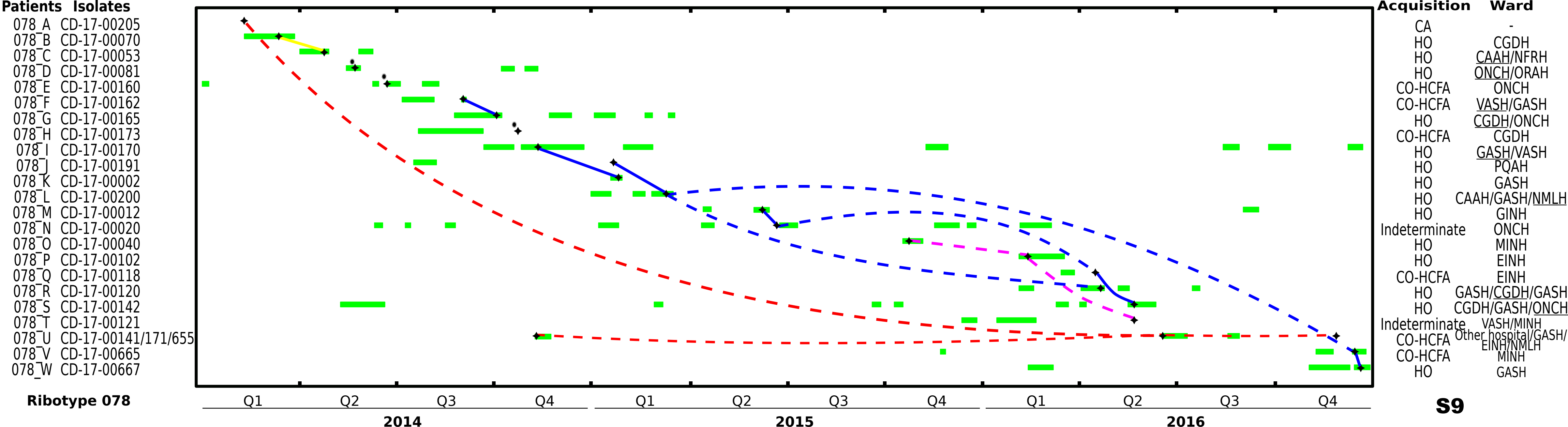
Patients Isolates
078_A CD-17-00205
078_B CD-17-00070
078_C CD-17-00053
078_D CD-17-00081
078_E CD-17-00160
078_F CD-17-00162
078_G CD-17-00165
078_H CD-17-00173
078_I CD-17-00170
078_J CD-17-00191
078_K CD-17-00002
078_L CD-17-00200
078_M CD-17-00012
078_N CD-17-00020
078_O CD-17-00040
078_P CD-17-00102
078_Q CD-17-00118
078_R CD-17-00120
078_S CD-17-00142
078_T CD-17-00121
078_U CD-17-00141/171/655
078_V CD-17-00665
078_W CD-17-00667
Ribotype 078
Q1 Q2 Q3 Q4 Q1 Q2 Q3 Q4 Q1 Q2 Q3 Q4
2014
2015
2016
Acquisition Ward
CA -
HO CGDH
HO CAAH/NFRH
HO ONCH/ORAH
CO-HCFA ONCH
CO-HCFA VASH/GASH
HO CGDH/ONCH
CO-HCFA CGDH
HO GASH/VASH
HO PQAH
HO GASH
HO CAAH/GASH/NMLH
HO GINH
Indeterminate ONCH
HO MINH
HO EINH
CO-HCFA EINH
HO GASH/CGDH/GASH
HO CGDH/GASH/ONCH
Indeterminate VASH/MINH
CO-HCFA Other hospital/GASH/EINH/NMLH
CO-HCFA MINH
HO GASH

S9

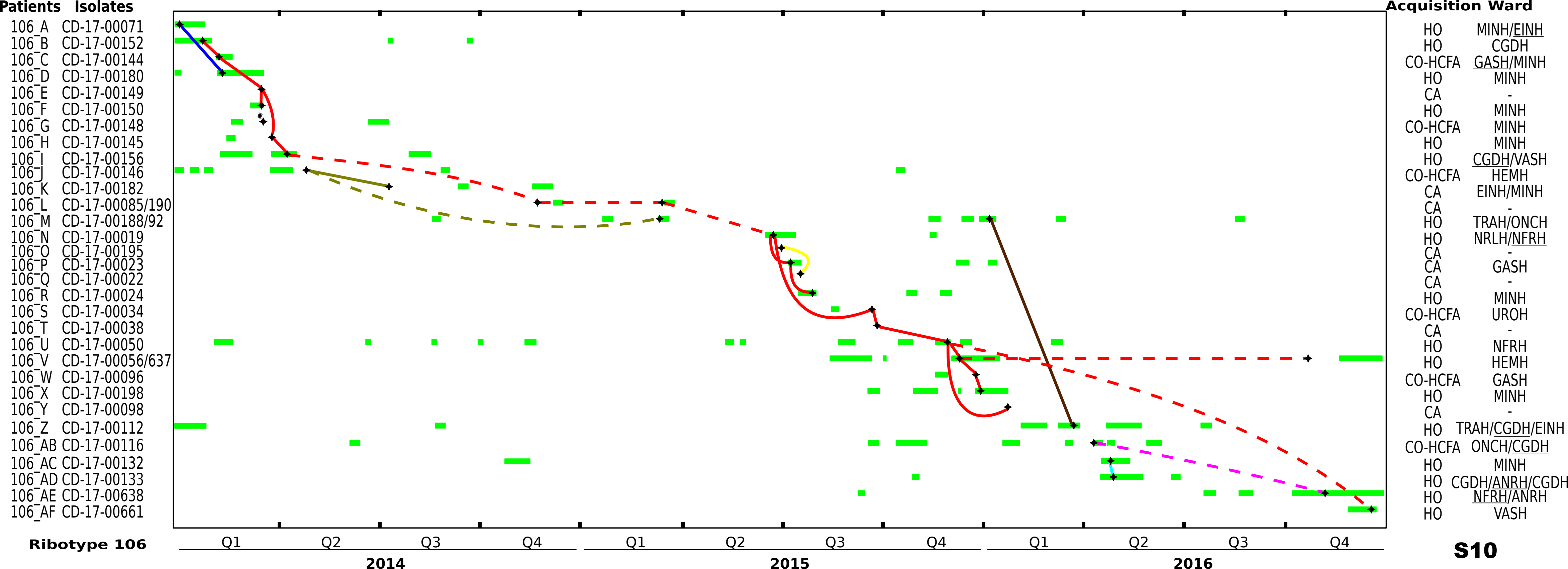
Patients Isolates
Acquisition Ward
106_A CD-17-00071 HO MINH/EINH
106_B CD-17-00152 HO CGDH
106_C CD-17-00144 CO-HCFA GASH/MINH
106_D CD-17-00180 HO MINH
106_E CD-17-00149 CA -
106_F CD-17-00150 HO MINH
106_G CD-17-00148 CO-HCFA MINH
106_H CD-17-00145 HO MINH
106_I CD-17-00156 HO CGDH/VASH
106_J CD-17-00146 CO-HCFA HEMH
106_K CD-17-00182 CA EINH/MINH
106_L CD-17-00085/190 CA -
106_M CD-17-00188/92 HO TRAH/ONCH
106_N CD-17-00019 HO NRLH/NFRH
106_O CD-17-00195 CA -
106_P CD-17-00023 CA GASH
106_Q CD-17-00022 CA -
106_R CD-17-00024 HO MINH
106_S CD-17-00034 CO-HCFA UROH
106_T CD-17-00038 CA -
106_U CD-17-00050 HO NFRH
106_V CD-17-00056/637 HO HEMH
106_W CD-17-00096 CO-HCFA GASH
106_X CD-17-00198 HO MINH
106_Y CD-17-00098 CA -
106_Z CD-17-00112 HO TRAH/CGDH/EINH
106_AB CD-17-00116 CO-HCFA ONCH/CGDH
106_AC CD-17-00132 HO MINH
106_AD CD-17-00133 HO CGDH/ANRH/CGDH
106_AE CD-17-00638 HO NFRH/ANRH
106_AF CD-17-00661 HO VASH
Ribotype 106
Q1 Q2 Q3 Q4
2014
Q1 Q2 Q3 Q4
2015
Q1 Q2 Q3 Q4
2016

S10

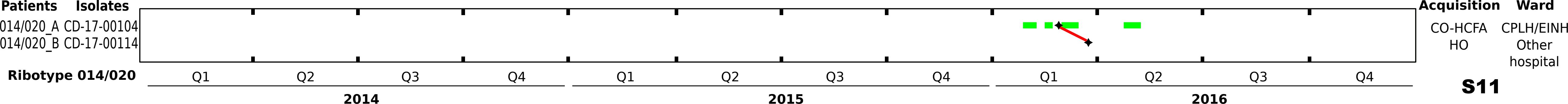
Patients
Isolates
014/020_A CD-17-00104
014/020_B CD-17-00114
Ribotype 014/020
Q1
Q2
Q3
Q4
Q1
Q2
Q3
Q4
Q1
Q2
Q3
Q4
2014
2015
2016
Acquisition
Ward
CO-HCFA
CPLH/EINH
HO
Other hospital
S11

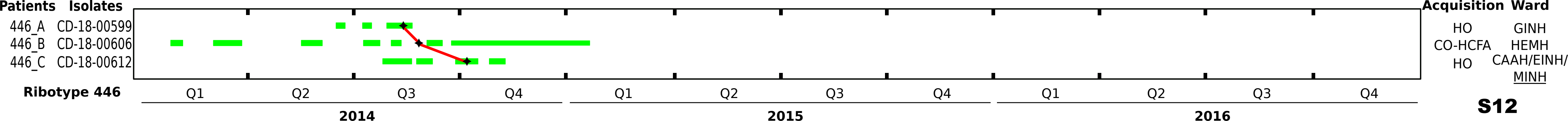

Patients
Isolates
446_A
CD-18-00599
446_B
CD-18-00606
446_C
CD-18-00612
Ribotype 446
Q1
Q2
Q3
Q4
Q1
Q2
Q3
Q4
Q1
Q2
Q3
Q4
2014
2015
2016
Acquisition
Ward
HO
GINH
CO-HCFA
HEMH
HO
CAAH/EINH/
MINH
S12

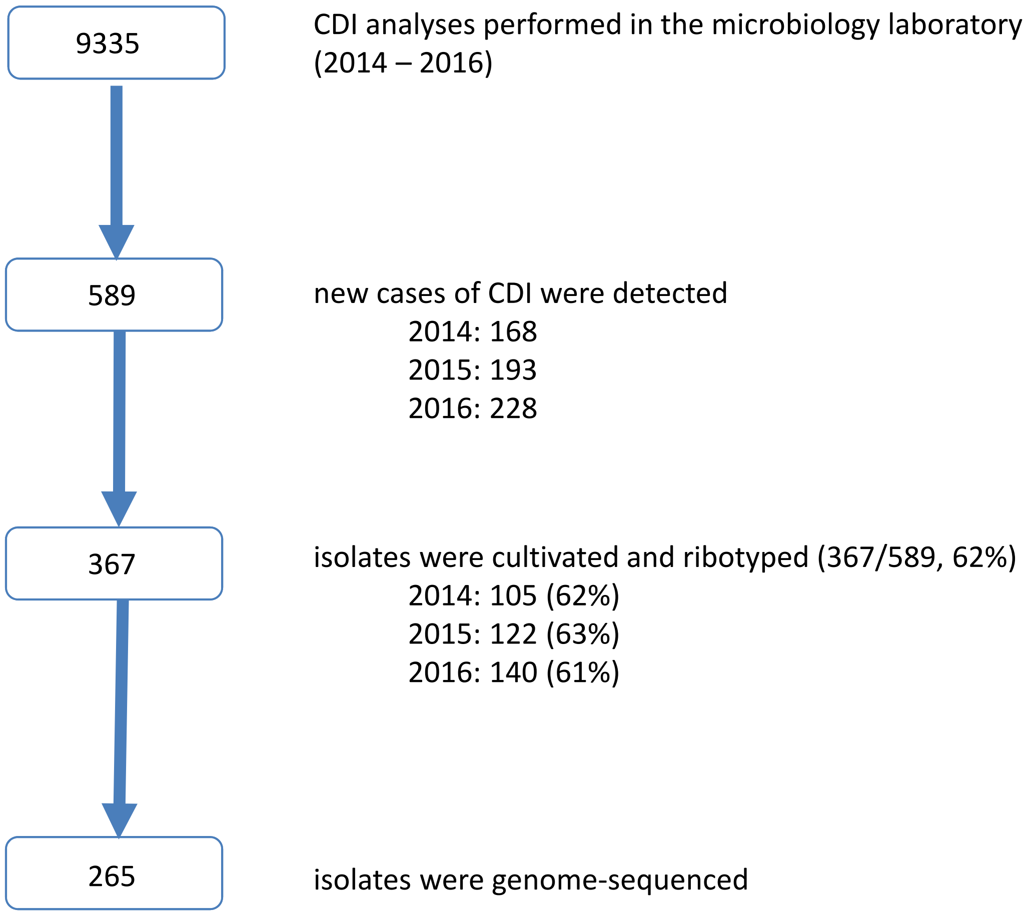
9335
CDI analyses performed in the microbiology laboratory (2014 – 2016)
589
new cases of CDI were detected
2014: 168
2015: 193
2016: 228
367
isolates were cultivated and ribotyped (367/589, 62%)
2014: 105 (62%)
2015: 122 (63%)
2016: 140 (61%)
265
isolates were genome-sequenced

S13
